# Palatal window osteotomy technique improves maxillary sinus augmentation in previously insufficient augmentation case

**DOI:** 10.1186/s40729-015-0018-y

**Published:** 2015-07-17

**Authors:** Daisuke Ueno, Takashi Kurokawa, Katsuichiro Maruo, Tsuneaki Watanabe, Jayanetti Asiri Jayawardena

**Affiliations:** 1 Department of Implantology and Periodontology, Kanagawa Dental University, Graduate School of Dentistry, 3-31-6 Tsuruya-cho, Kanagawa-ku Yokohama, Japan; 2 Unit of Oral and Maxillofacial Implantology, Tsurumi University Dental Hospital, Yokohama, Japan; 3 Department of Removal Prosthodontics, Kanagawa Dental University, Graduate School of Dentistry, Yokosuka, Japan; 4 Department of General Education, Tsurumi University, School of Dental Medicine, Yokohama, Japan

**Keywords:** Sinus floor augmentation, Dental implants, Surgical technique, Palatal osteotomy

## Abstract

**Introduction:**

Perforation of the Schneiderian membrane is the most common complication in sinus floor augmentation (SFA). When volume of grafting is qualified to prevent enlargement of the membrane perforation, lack of bone volume may occur in optimal site.

**Case presentation:**

SFA was performed in sites #24 to 26 in a 63-year-old male. However, a 10-mm size perforation of the Schneiderian membrane occurred in site #26. Although the sinus cavity was grafted with deproteinized bovine bone mineral (DBBM) after repair of membrane perforation, insufficient bone formation was observed on palatal and distal aspects of site #26 at 5 months after SFA. Although additional SFA was required for implant placement, it seemed to be difficult to elevate the membrane by a conventional lateral approach in the palatal aspect of the sinus floor (site #26). Considering the configuration of new bone formation, it was decided to perform the palatal antrostomy approach. The Schneiderian membrane was elevated without perforation, and the sinus cavity was grafted with DBBM mixed with venous blood. Two 12-mm long, 4.1-mm diameter implants were placed in sites #14 and 16. Four months after implant placement, abutment-connection surgery was successfully performed. The radiographic image indicated improved radiopacity, without obvious bone resorption in site #26.

**Conclusion:**

The palatal window osteotomy technique could be considered as an alternative method for augmentation of maxillary sinus in cases where difficulty is encountered to elevate a membrane by a conventional approach (e.g., in cases in which buccal bone height is long).

## Introduction

Sinus floor augmentation (SFA) is the most common technique to obtain bone height for implant placement in posterior maxilla. The most common method is the classical lateral antrostomy approach; After raising a full-thickness flap on the buccal side of the alveolar ridge, a trap door is created by a round bur [1]. The sinus membrane is dissected, and the trap door is rotated medially to push the Schneiderian membrane apically. Then the graft material is placed on the sinus floor. Although a conventional lateral window technique is known to be very predictable with good long-term success, only few reports have been introduced to evaluate the palatal antrostomy approach [2, 3]. The authors reported slight usability such as postoperative comfort compared to conventional buccal antrostomy approach [3]. This case demonstrates significant bone augmentation using a palatal antrostomy technique in the palatal aspect of the sinus floor which makes it difficult to elevate the Schneiderian membrane by a conventional approach.

## Clinical report

**Fig. 1:**
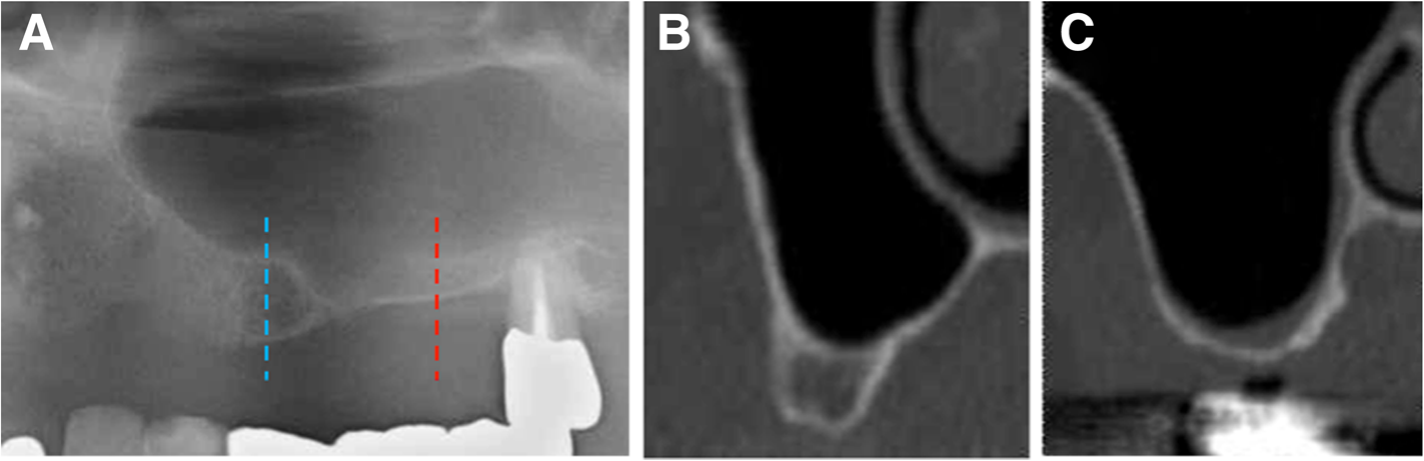
Preoperative radiographic images. **a** Orthopantomography shows that inadequate bone height was observed in sites #24 (*blue line*) and #26 (*red line*). **b** Coronal CT image of the *blue line*; vertical bone height is 4 mm. **c** Coronal CT image of the *red line*; vertical bone height is only 1 mm

A 63-year-old male patient was introduced to the Unit of Oral and Maxillofacial Implantology, Tsurumi University Dental Hospital for implant treatment in April 2013 (Fig. 1). Since vertical bone heights in sites # 24 (4 mm) and 26 (less than 1 mm) were not enough for implant placement, SFA was performed prior to implant placement. However, a 10-mm size perforation of the Schneiderian membrane occurred in site #26 during sinus floor elevation. The membrane perforation was covered with a sheet-like collagen sponge^1^. Then, the sinus cavity was grafted with deproteinized bovine bone mineral (DBBM)^2^ mixed with venous blood [4]. Use of DBBM was approved by the ethical committee of Tsurumi University. Nevertheless, volume of grafting in the posterior area was qualified to prevent enlargement of the membrane perforation. Site #24 was augmented with guided bone regeneration to increase bone width for implant placement.

**Fig. 2:**
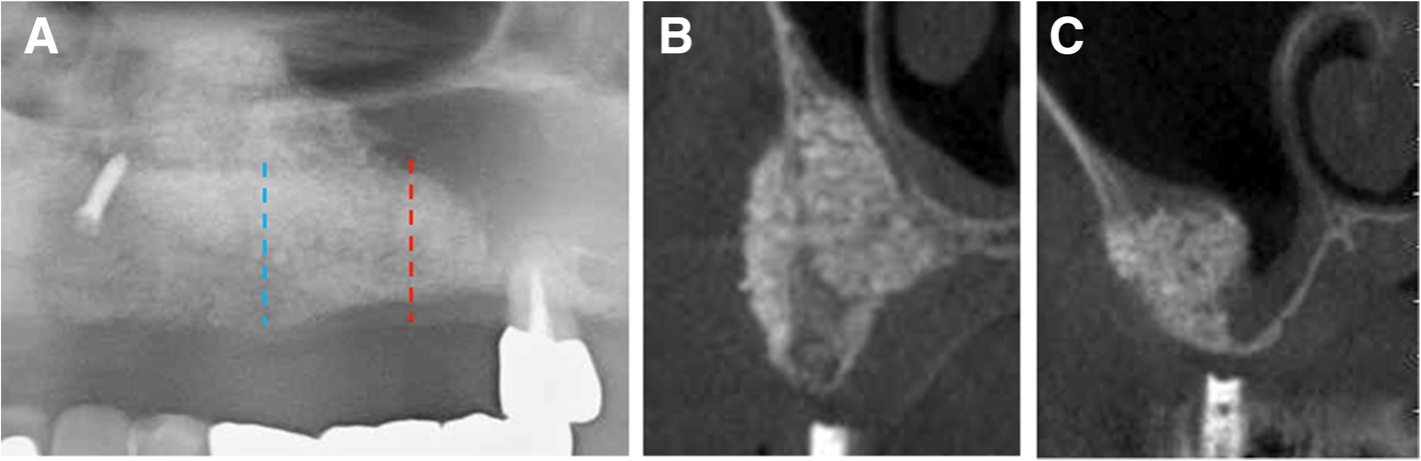
Radiographic image, 5 months after sinus bone augmentation. **a** Orthopantomography shows inadequate bone height observed in sites #26 (*red line*). **b** Coronal CT image of the *blue line*; vertical bone height is 15 mm. Horizontal augmentation is also achieved. **c** Coronal CT image of the *red line*; no bone augmentation was achieved in the palatal aspect of sinus cavity

No complications, such as postoperative nose bleeding, occurred during the healing period. Five months after sinus floor augmentation, a cone beam computed tomography (CBCT) image showed that an adequate volume of bone augmentation was achieved in site #24 (Fig. 2). In contrast, lack of bone formation was observed in palatal and distal aspects of site #26. Therefore, additional bone augmentation was planned in order to place implants with optimal position and direction.

**Fig. 3:**
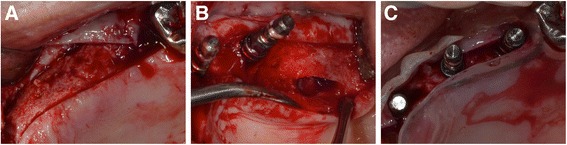
Sinus bone augmentation with implant placement after palatal window osteotomy. After pala-crestal incision (**a**) and raising full-thickness flaps on the buccal and palatal sides of the alveolar ridge, palatal antrostomy is performed by a round bur (**b**). An implant was placed in optimal position after bone grafting in the palatal aspect of sinus cavity (**c**)

A pala-crestal incision performed from site #21 to the interproximal aspects of the #27. The incision was extended intrasulcaly, and vertical release incisions were made at the mesiobuccal angle of the maxillary pad (Fig. 3). Full-thickness flaps were elevated buccal and palatal aspects. The palatal wall of the maxillary sinus was exposed after the elevation of a mucoperiosteal flap. A piezoelectric device^3^ and diamond-coated round bur was used with copious saline irrigation to create the palatal window of the maxillary sinus. Then, the Schneiderian membrane was elevated by sinus lift elevator without perforation (Fig. 3). Since the palatal vault is steep, and palatal bone is thick, Tinti sinus lift elevator^4^ which can be bent hard was used to separate the palatal Schneiderian membrane. Then, Memmingen sinus lift elevator^5^ was used to elevate the membrane apically. After the sinus cavity was grafted with DBBM^2^ mixed with venous blood, two 12-mm long, 4.1-mm diameter implants^6^^,^^7^ were placed in sites #24 and 26 (Fig. 3). Then, further bone augmentation was performed on the buccal implant from the palatal bony window. The insertion torques of implants #24 and 26 were 35 and 25 Ncm, respectively. The graft sites were covered with a sheet-like collagen sponge to improve graft stability^1^. After periosteal-releasing incisions, flaps were sutured without flap tension. The post-surgical course was uneventful.

**Fig. 4:**
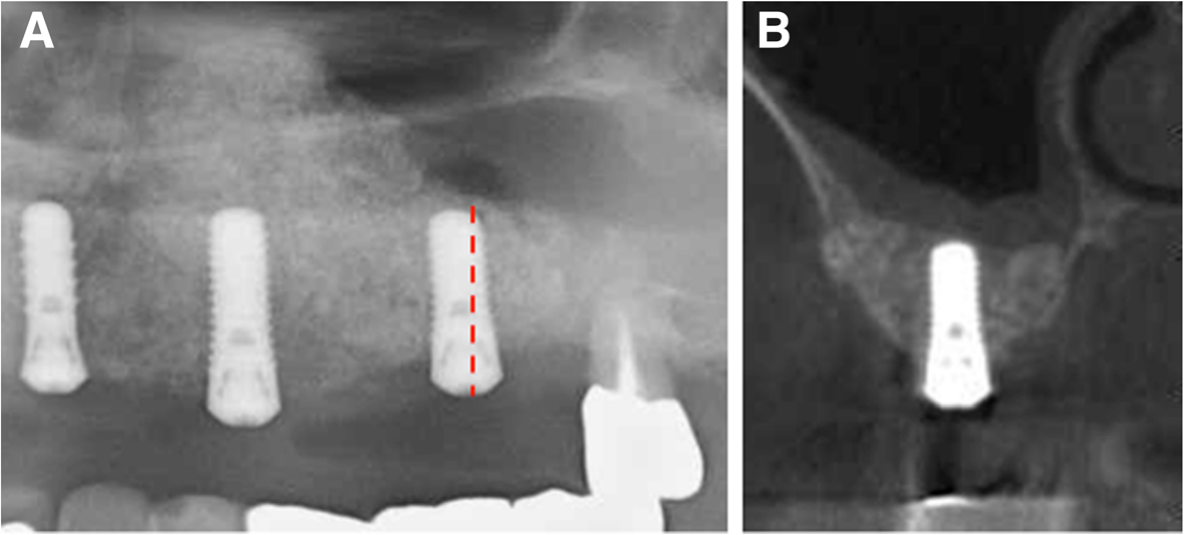
Radiographic image. Four months following 2nd sinus floor augmentation with implant placements. **a** Significant bone augmentation is achieved in site #26. **b** Coronal CT image of the *red line* shows that sufficient bone support with improved radiopacity was achieved in the palatal aspect of sinus cavity

Four months after implant placement, abutment-connection surgery was performed. The radiographic image improved radiopacity without obvious bone resorption in the optimal site (Fig. 4). The values of the implant stability quotient (ISQ) of the implants in #24 and 26 were 78 and 71, respectively. All implants were functioning well 6 months after occlusal loading.

## Discussion

The palatal window osteotomy technique is previously described as beneficial because it has a higher postoperative comfort, especially for edentulous patients, because full dentures could be incorporated directly after surgery with an almost perfect fit [3, 5]. As another advantage, this case demonstrated significant bone augmentation in the palatal aspect of the sinus floor which makes it difficult to elevate membrane by conventional approach.

Perforation of the sinus membrane is the most common intra-operative complication in maxillary sinus floor augmentation. According to a systematic review, mean prevalence of membrane perforation was 19.5 % [6]. Membrane perforation was usually closed by fibrin glue, suturing or, covering them with a collagen membrane. Depending on the size and location of the perforation, a sufficient quantity of bone augmentation is not possible in the optimal site. In cases of larger perforations, discontinuation of SFA, and reoperation after healing of the sinus membrane may be a more reliable method. However, the staged recovery approach requires an additional treatment period. In the present case, bone volume required for implant placement was supplemented by the first SFA. Additional SFA with the palatal window osteotomy technique was able to graft in palatal sinus cavity which is the insufficient bone volume area. A particular advantage of the palatal window osteotomy is that it can easily approach the cavity compared to buccal window osteotomy, when buccal bone in maxillary sinus is thick and long. From these findings, it may be suggested that maxillary sinus augmentation with the palatal window osteotomy approach is useful in compensation of the palatal sinus cavity. Caution should be exercised during elevation of the palatal flap and osteotomy preparation in the palatal wall to avoid damage to the greater palatine neurovascular bundle (GPB) which contains greater palatine artery, vein, and nerve. Since GPB exits through the greater palatine foramen and runs anteriorly in the bone groove of the palate, three-dimensional analysis using CBCT or CT would reduce surgical complications [7]. Further studies are needed to confirm the efficiency of the palatal window osteotomy technique.

## Consent

Written informed consent was obtained from the patient for publication of this report and any accompanying images.

## Footnotes

1 TERUDERMIS, Orinpus-Termo Biomaterials, Tokyo, Japan.

2 Bio-Oss®, Geistlich, Biomaterials, Wolhuser, Switzerland.

3 Sonic Surgeon 300, Morita Corporation, Tokyo, Japan.

4 Stoma Tinti Sinus Lift Elevator, Stoma, Germany.

5 Stoma Memmingen Sinus Lift Elevator, Stoma, Germany.

6 Straumann® Bone Level Implant, Basel, Switzerland.

7 Straumann® Tapered Effect Implant, Basel, Switzerland.
